# Disparate molecular mechanisms in cardiac ryanodine receptor channelopathies

**DOI:** 10.3389/fmolb.2024.1505698

**Published:** 2024-12-24

**Authors:** Yadan Zhang, Monika Seidel, Camille Rabesahala de Meritens, Astrid Beckmann, Syeda Ahmed, Melanie Hurtz, F. Anthony Lai, Esther Zorio, Dimitris Parthimos, Spyros Zissimopoulos

**Affiliations:** ^1^ Swansea University Medical School, Institute of Life Science, Swansea, United Kingdom; ^2^ College of Medicine and Biomedical Research Centre, Qatar University, Doha, Qatar; ^3^ Inherited Cardiac Disease Unit, Hospital Universitario y Politécnico La Fe, Valencia, Spain; ^4^ CAFAMUSME Research Group, Instituto de Investigación Sanitaria La Fe, Valencia, Spain; ^5^ Medicine Department, Universitat de València, Valencia, Spain; ^6^ Research group CB16/11/00261, Center for Biomedical Network Research on Cardiovascular Diseases (CIBERCV), Madrid, Spain; ^7^ School of Medicine, Division of Cancer and Genetics, Cardiff University, Cardiff, United Kingdom

**Keywords:** arrhythmia, calcium cycling, excitation-contraction coupling, intracellular calcium channel, ryanodine receptor

## Abstract

**Aims:**

Mutations in the cardiac ryanodine receptor (RyR2) are associated with catecholaminergic polymorphic ventricular tachycardia (CPVT). This study investigates the underlying molecular mechanisms for CPVT mutations within the RyR2 N-terminus domain (NTD).

**Methods and Results:**

We consulted the high-resolution RyR2 structure in both open and closed configuration to identify mutations G357S/R407I and A77T, which lie within the NTD intra- and inter-subunit interface with the Core Solenoid (CSol), respectively. Their structural and functional roles were compared to R169L, a mutation that lies within the NTD-NTD inter-subunit interface. Using chemical cross-linking and co-immunoprecipitation assays, we show that R169L disrupts NTD tetramerization, while it does not alter the NTD-CSol interaction. Single cell Ca^2+^ imaging revealed that R169L increases the number of spontaneous Ca^2+^ transients and the proportion of oscillating cells, while reducing the Ca^2+^ store content. G357S and R407I do not affect NTD tetramerization, but they also do not alter the NTD-CSol interaction. Functionally, RyR2^G357S^-expressing cells have Ca^2+^ handling properties similar to RyR2^WT^. A77T enhances the NTD-CSol interaction, while it does not affect NTD tetramerization. Like R169L, A77T also increases the number of spontaneous Ca^2+^ transients and the proportion of oscillating cells, and it reduces the Ca^2+^ store content. However, unlike R169L that displays Ca^2+^ transients of normal amplitude and shorter duration, Ca^2+^ transients for A77T are of smaller amplitude and normal duration.

**Conclusion:**

The NTD-CSol inter-subunit interface variant, A77T, produces a hyperactive channel by altering a different structure-function parameter to other CPVT mutations within the RyR2 NTD. Reduced NTD-NTD inter-subunit interaction and reinforced NTD inter-subunit interaction with CSol are distinct molecular mechanisms for gain-of-function RyR2 arrhythmogenic mutations.

## 1 Introduction

**FIGURE 1:**
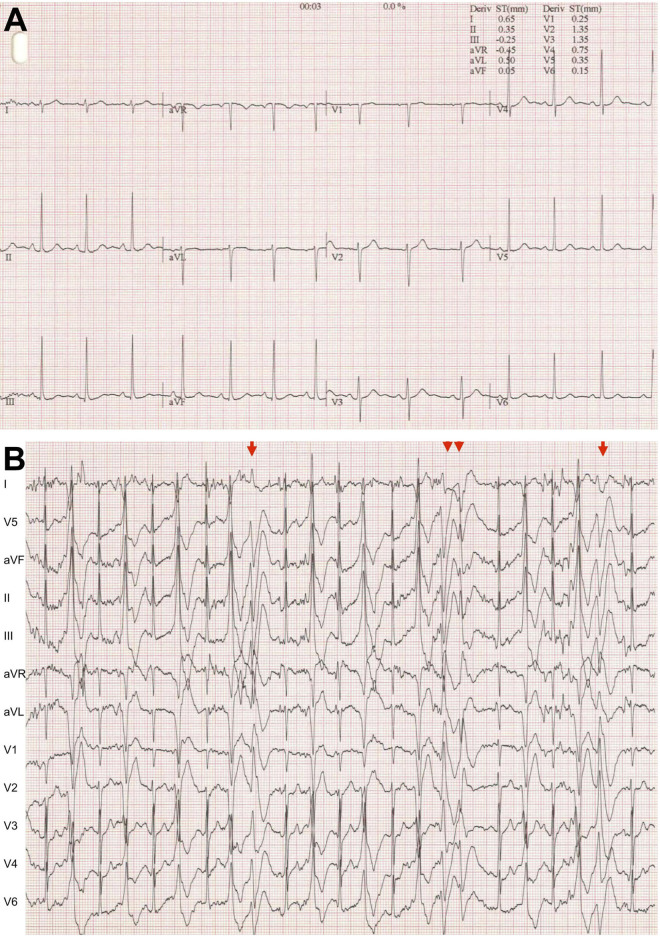
Typical CPVT electrocardiogram **(A).** At rest. **(B)** During exercise testing. Very frequent ventricular extrasystoles, biderectional ventricular couplets (arrow) and non-sustained ventricular tachycardia (double arrowheads).

The ryanodine receptor (RyR2) plays a vital role in cardiac excitation-contraction coupling by mediating sarcoplasmic reticulum Ca^2+^ release. Abnormal RyR2 function resulting in aberrant cardiomyocyte Ca^2+^ handling leads to arrhythmias and sudden death. To date, around 350 missense mutations have been identified in RyR2, most linked with catecholaminergic polymorphic ventricular tachycardia (CPVT) (Venetucci et al., 2012; Fowler and Zissimopoulos, 2022). CPVT, a relatively common disease estimated to affect one in 10,000 people, is triggered by emotional or physical stress and exhibits a mortality rate of >30% if left untreated (Leenhardt et al., 2012; Napolitano et al., 2014; Priori et al., 2021). Resting electrocardiogram is usually normal and patients develop ventricular arrhythmias during exercise testing (Figure 1). Recently, several hypoactive RyR2 mutations have been described, which are associated with the calcium release deficiency syndrome (Tester et al., 2020; Sun et al., 2021). In addition, RyR2 mutations have been implicated in intellectual disability (Lieve et al., 2018) and genetic generalized epilepsy (Yap and Smyth, 2019), which is not surprising given that RyR2 is expressed in the brain (Torres and Hidalgo, 2023).

The functional RyR2 channel, consisting of four identical subunits of ∼5,000 amino acids, is organized in discreet structural domains. The transmembrane domain encompassing the Ca^2+^ permeable pore is located at the C-terminus (residues 4484-4886). The Core Solenoid (CSol, residues 3612-4206) has been described as the gatekeeper of the channel because it is directly associated with the pore-forming region (des Georges et al., 2016; Peng et al., 2016). The N-terminus domain (NTD, residues 1-640), although distant from the pore in terms of both primary and tertiary structure, is essential for RyR2 function regulating both channel opening and closing (Tang et al., 2012; Zissimopoulos et al., 2013; Liu et al., 2015; Seidel et al., 2015; Faltinova et al., 2017; Seidel et al., 2021). The NTD’s dual role in channel gating likely arises from its involvement in separate RyR2 inter-domain interactions due to the extensive and intricate folding of the RyR2 polypeptide chain. Indeed, the high-resolution 3D structures of the RyR1/2 homotetrameric channels (des Georges et al., 2016; Peng et al., 2016) have revealed that the NTD forms an interface with a neighboring NTD (referred to as type I hereafter) and separate intra- and inter-subunit interfaces (referred to as type II^intra^ and II^inter^, respectively) with the CSol.

The type II residues that are within 6Å distance from each other and therefore likely to directly participate in RyR2 intra- and inter-subunit NTD-CSol interactions are given in Table 1. RyR2 NTD mutations residing within different inter-domain interfaces are potentially affecting channel function by separate mechanisms. To test this hypothesis, we biochemically and functionally characterized mutations A77T, R169L and G357S, representative type II^inter^, I, and II^intra^ variants, respectively.

**TABLE 1:** RyR2 residues at the NTD-CSol inter- and intra-subunit interfaces Atomic distance between listed residues taken from the cryo-electron microscopy structure of RyR2 in the closed (5GO9) and open state (5GOA) (Peng et al., 2016).

RyR2 NTD-CSol inter-subunit interface			RyR2 NTD-CSol intra-subunit interface		
Residues	Closed state	Open state	Residues	Closed state	Open state
S74 – W3890	5.6Å	6.1Å	Q225 – T3862	4.0Å	5.1Å
R76 – L3805	4.4Å	3.9Å	Q225 – G3863	4.9Å	4.1Å
R76 – E3809	5.1Å	5.6Å	K355 – T3867	5.7Å	7.8Å
R76 – D3887	6.0Å	4.2Å	G357 – N3865	6.8Å	5.7Å
R76 – W3890	3.6Å	3.0Å	G357 – T3867	5.2Å	7.3Å
R76 – Y3891	4.9Å	5.5Å	D358 – T3867	6.8Å	4.2Å
R76 – S3893	5.8Å	5.7Å	R407 – N3865	3.3Å	5.8Å
A77 – W3890	4.2Å	4.3Å	Q409 – N3865	4.2Å	5.5Å
E80 – W3890	6.2Å	4.7Å	H410 – N3865	5.8Å	9.2Å
R111 – N4008	6.4Å	3.6Å	H472 – S3672	5.7Å	6.5Å
T112 – E4005	5.8Å	6.0Å	H472 – R3673	3.5Å	4.1Å
E171 – L3879	5.4Å	4.8Å	H472 – T3677	4.6Å	5.2Å
E171 – E3883	4.0Å	4.3Å	E473 – L3676	3.6Å	3.7Å
E171 – D3887	5.2Å	8.2Å	E473 – Y3780	5.3Å	5.7Å
G172 – E3883	3.7Å	3.4Å	E473 – K3784	3.7Å	3.4Å
E173 – R3939	4.9Å	5.1Å	E473 – D3786	5.6Å	7.0Å
K174 – D3942	4.4Å	3.9Å	Q476 – L3676	3.1Å	4.1Å
K174 – E4005	3.2Å	5.7Å	Q476 – T3677	5.2Å	5.1Å
			Q476 – E3678	2.4Å	4.0Å
			N477 – K3784	6.6Å	5.9Å
			L479 – E3678	4.8Å	5.2Å
			R480 – E3678	3.1Å	3.1Å
			K483 – E3678	5.8Å	4.8Å

## 2 Methods

### 2.1 Materials

The human embryonic kidney (HEK) 293 cell line was obtained from ATCC® (CRL-1573), mammalian cell culture reagents from Thermo Scientific, Cal-520 a.m. from Stratech, protease inhibitor cocktail (Complete™) from Roche, nProtein-A Sepharose from GE Healthcare, electrophoresis equipment and reagents from Bio-Rad, and the enhanced chemiluminescence detection kit from Thermo Scientific. Mouse anti-cMyc (9E10) was from Santa Cruz Biotechnology, mouse anti-HA (16B12) from Biolegend, rabbit anti-HA (ab9110) and goat anti-mouse IgG conjugated with horseradish peroxidase from Abcam, DNA restriction endonucleases from New England Biolabs, Pfu DNA polymerase from Promega, site-directed mutagenesis kit (QuikChange II XL) from Agilent Technologies, CHAPS, normal rabbit IgG, oligonucleotides and all other reagents from Merck unless otherwise stated.

### 2.2 Plasmid construction

The plasmids encoding for wild-type RyR2 N-terminus (NT, residues 1-906) tagged with the cMyc epitope, and RyR2 C-terminus (HA-RyR2-CT, residues 3529-4967) tagged with the HA epitope have been described previously (Zissimopoulos et al., 2013; Seidel et al., 2021). Desired missense mutations (A77T, R169L, G357S, R407I) were generated using the site-directed mutagenesis QuikChange II XL kit and appropriate primers as recommended by the supplier. Plasmids encoding for human RyR2^A77T^, RyR2^R169L^ and RyR2^G357S^ were prepared by replacing a SpeI–BstEII ∼3.8 kb DNA fragment into the WT plasmid. All plasmid constructs were verified by direct DNA sequencing.

### 2.3 Chemical cross-linking

HEK293 cells were transiently transfected using TurboFect (Thermo Scientific) according to the provider’s instructions. 24 h post-transfection, cells were homogenized on ice in buffer (5 mM HEPES, 0.3 M sucrose, 10 mM DTT, pH 7.4) by 20 passages through a needle (0.6 × 30 mm) and dispersing the cell suspension through half volume of glass beads (425–600 microns). Cell homogenate free of nuclei and heavy protein aggregates was obtained by centrifugation at 1500 *g* for 5 min at 4°C, followed by a second centrifugation step at 20,000 x *g* for 10 min at 4°C. Cell homogenate supernatant (20 μg) was incubated with glutaraldehyde (0.0025% or 260 μmol/L) for the following time-points: 0, 2, 5, 10, 15, 20, 30 and 60 min. The reaction was stopped with the addition of hydrazine (2%) and SDS-PAGE loading buffer (60 mM Tris, 2% SDS, 10% glycerol, 5 mM EDTA, 0.01% bromophenol blue, pH 6.8). Samples were analyzed by SDS-PAGE and Western blotting with Ab^cMyc^ (9E10, 1:1000 dilution). Tetramer to monomer ratio was determined by densitometry using a GS-900 Scanner (Bio-Rad) and Image Lab software (Bio-Rad). Tetramer formation was calculated as follows: T = OD_T_/(OD_T_ + OD_M_)x100, where OD_T_ and OD_M_ correspond to optical density obtained for tetramer and monomer bands, respectively. Statistical analysis was carried out with GraphPad Prism software.

### 2.4 Co-immunoprecipitation

HEK293 cells were transiently co-transfected with plasmid DNA for HA-RyR2-CT together with cMyc-RyR2-NT constructs using TurboFect. Cells were homogenized on ice 24 h post-transfection, in buffer (20 mM Tris, 150 mM NaCl, pH 7.4) as described in 2.3 above. Cell nuclei and glass beads were removed by centrifugation at 1500 *g* for 5 min at 4°C and the supernatant was incubated for 1 h at 4°C in the presence of 0.5% CHAPS under rotary agitation. Following solubilization and centrifugation at 20,000 *g* for 10 min at 4°C to remove the insoluble material, the supernatant was incubated at 4°C for 2 h with Protein A-Sepharose beads (GE Healthcare) and 1 μg of Ab^HA^ (rabbit ab9110) under rotary agitation (1 μg of normal, non-immune rabbit IgG was used as negative control). Beads were recovered at 1500 x *g* for 2 min at 4°C, washed two times with IP buffer (20 mM Tris, 150 mM NaCl, 0.5% CHAPS, pH 7.4) and proteins were eluted with SDS-PAGE loading buffer. A small amount (1/10th) of the IP samples was analyzed by SDS-PAGE and Western blotting with Ab^HA^ (16B12, 1:1000 dilution) to assess HA-RyR2-CT expression and immunoprecipitation. The rest (9/10th) of the IP samples were analyzed by SDS-PAGE and Western blotting with Ab^cMyc^ (9E10, 1:500 dilution) to assess the amount of the co-precipitated RyR2 NT construct. The amount of co-precipitated RyR2 NT proteins was determined by densitometry (using GS-900 Scanner and Image Lab software), normalized against the amount of input protein in the lysate and specific binding was calculated by subtracting the non-immune IgG IP signal from the anti-HA IP signal. Statistical analysis was carried out with GraphPad Prism software.

### 2.5 Calcium imaging

HEK293 cells (∼1 x 10^5^) were seeded on poly-lysine coated glass bottom dishes (MatTek) and transiently transfected with plasmid DNA for full-length human RyR2 using Effectene (Qiagen) according to the manufacturer’s instructions. After 48 h, cells were loaded with Cal-520 a.m. (8 μM) for 1 h at 37°C and immersed in buffer (120 mM NaCl, 25 mM HEPES, 5.5 mM glucose, 4.8 mM KCl, 1.3 mM CaCl_2_, 1.2 mM KH_2_PO_4_, 1.2 mM MgCl_2_, pH 7.4) for imaging at 37°C. RyR2-mediated spontaneous Ca^2+^ release events were monitored using a laser scanning confocal microscope (Leica SP5) and LAS-AF software (Leica Microsystems) with the following parameters: ×20 magnification objective lens, excitation at 488 nm and fluorescence emission detected at 500–550 nm, 512 x 512 pixel resolution, 100 msec time interval and scanning speed of 400 Hz. Cells were imaged for 5 min and challenged with 10 mM caffeine after ∼4½ min. Acquired regions of interest representing global Ca^2+^ environments (typically ∼50 μm^2^) were selected. A broad range of parametric values was calculated from experimental traces by in house developed MATLAB (MathWorks) based software. Parameters include spontaneous Ca^2+^ transient amplitude and duration, number of Ca^2+^ transient events, caffeine-induced Ca^2+^ transient amplitude (taken as indication of Ca^2+^ store content) and proportion of oscillating cells (number of cells displaying spontaneous Ca^2+^ release events relative to the total number of cells responding to caffeine). Statistical analysis was carried out with GraphPad Prism software.

## 3 Results

### 3.1 A77T and R169L affect distinct RyR2 structure parameters

**FIGURE 2:**
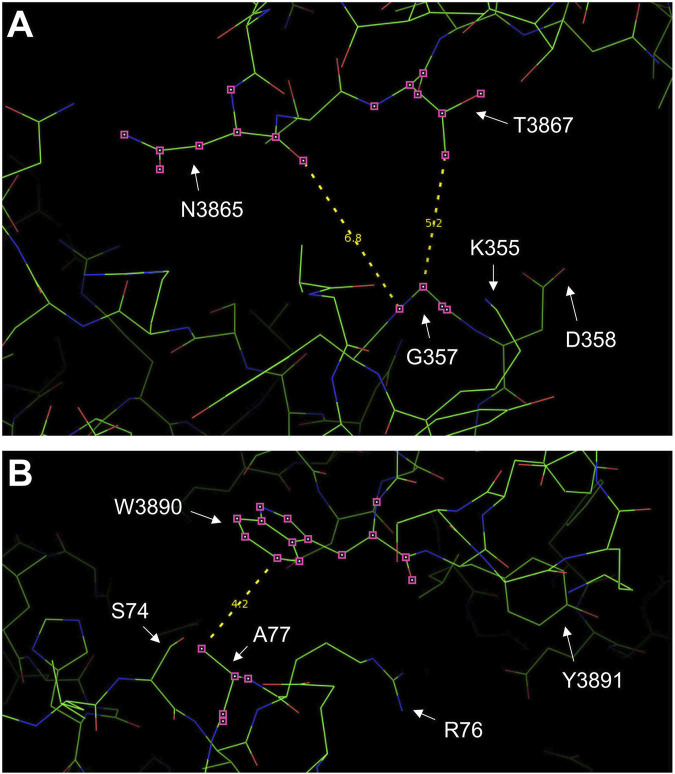
RyR2 residues at the NTD-CSol intra- and inter-subunit interfaces. Images of the NTD-CSol intra- and inter-subunit interfaces generated using PyMol from the RyR2 closed state (5GO9) structure. **(A)** The locations of residue G357 that is in close apposition to residues N3865 and T3867 on the same subunit, and the G357-N3865 and G357-T3867 distances (in Å) are depicted. **(B)** The locations of residue A77 on one subunit that is in close apposition to residue W3890 on the neighboring subunit and the A77-W3890 distance (in Å) are depicted.

We chose to study the R169L type I mutation (Ohno et al., 2015) specifically because it resides within the β8-β9 loop (residues 165-179), which is the primary determinant for RyR2 N-terminus self-association and a key element for channel function (Seidel et al., 2021; Zhang et al., 2022). G357S (Medeiros-Domingo et al., 2009) was selected because it resides within the NTD-CSol intra-subunit contact (Table 1; Figure 2A). A77T was chosen because it lies within the NTD-CSol inter-subunit contact (Table 1; Figure 2B), and it has been implicated in cardiac and neuronal disorders (Yap and Smyth, 2019).

**FIGURE 3:**
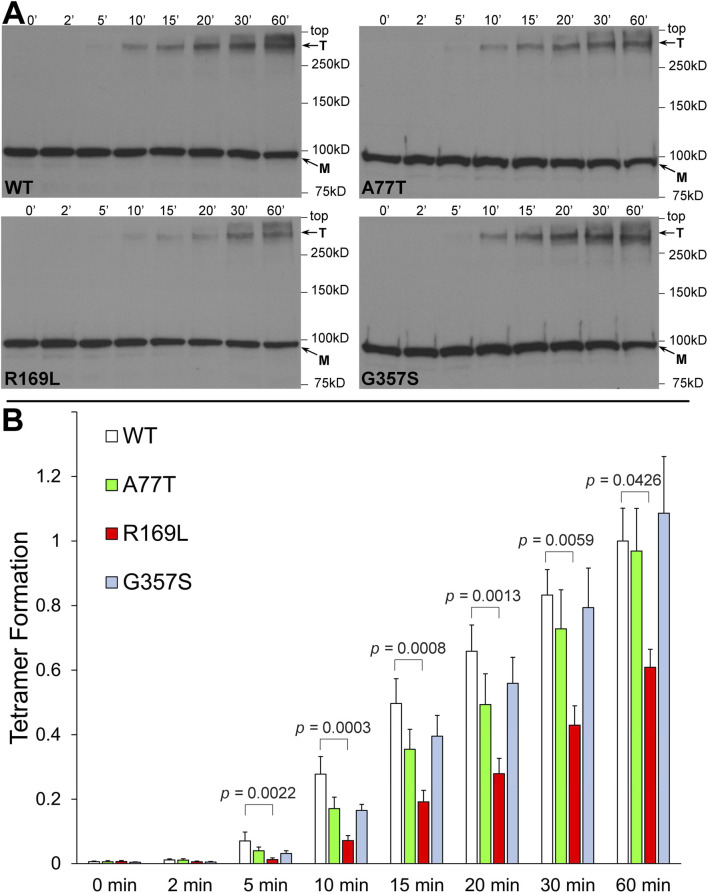
A77T, R169L and G357S differentially affect RyR2 N-terminus tetramerization. Chemical cross-linking assays of HEK293 cell homogenates expressing NT^WT^ (RyR2 residues 1-906, cMyc-tagged) or pro-arrhythmic mutants, NT^A77T^, NT^R169L^, NT^G357S^. **(A)** Cell homogenates were incubated with glutaraldehyde for the indicated time points and analyzed by SDS-PAGE (6% gels) and Western blotting using Ab^cMyc^; monomer (M) and tetramer (T) are indicated with the arrows. **(B)** Densitometric analysis was carried out on the bands corresponding to tetramer and monomer moieties and used to calculate tetramer formation. Data (n ≥ 8) are normalized for WT and given as mean value ±SEM; statistical analysis was carried out using one-way Anova with Dunnett’s multiple comparisons test.

RyR2 NTD tetramerization was assessed by chemical cross-linking. NT^WT^ (human RyR2 residues 1-906, cMyc-tagged), NT^A77T^, NT^R169L^ and NT^G357S^ were expressed in HEK293 cells and reacted with glutaraldehyde, which creates stable covalent chemical bridges between pre-existing protein complexes, and tetramer formation was analyzed by Western blotting using Ab^cMyc^ (Figure 3). Time-dependent formation of a ∼400 kDa band indicating the existence of a tetrameric assembly of ∼100 kDa RyR2-NT protomers (Zissimopoulos et al., 2013) was evident for WT and mutants. As we have recently reported for the R169Q variant (Zhang et al., 2022), the type I R169L mutation significantly reduced tetramer formation compared to WT. In contrast, the type II variants A77T and G357S, which are located away from the NTD-NTD interface, produced tetramers comparable to WT.

**FIGURE 4:**
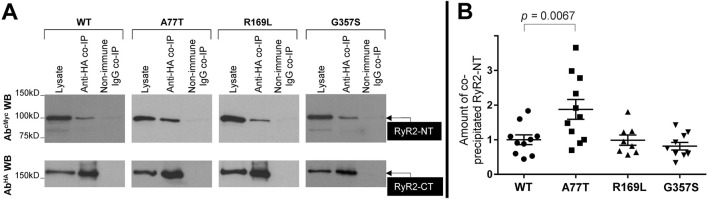
A77T, R169L and G357S differentially affect RyR2 N-terminus interaction with C-terminus. Co-immunoprecipitation assays from HEK293 cells co-expressing NT^WT^ or pro-arrhythmic mutants, NT^A77T^, NT^R169L^, NT^G357S^, together with HA-tagged RyR2-CT (residues 3529-4967). **(A)** HA-RyR2-CT was immunoprecipitated with Ab^HA^ from CHAPS-solubilized cell lysates and the presence of co-precipitated NT^WT^/NT^A77T^/NT^R169L^/NT^G357S^ was analyzed by SDS-PAGE (6% gels) and Western blotting using Ab^cMyc^ (top). To detect immuno-isolated HA-RyR2-CT, 1/10th of IP samples was analyzed by Western blotting using Ab^HA^ (bottom). Non-immune rabbit IgG served as negative control (non-specific binding). An aliquot of HEK293 cell lysate corresponding to 1% of the amount processed in the co-IP assay was included in the gels to assess protein expression. **(B)** Data summary for NT specific binding (non-immune IgG IP signal subtracted from anti-HA IP signal) following densitometric analysis and normalization to each construct’s respective lysate. Data (n ≥ 8) are normalized for WT and given as mean value ±SEM; statistical analysis was carried out using one-way Anova with Dunnett’s multiple comparisons test.

To assess NTD-CSol interactions, we conducted co-immunoprecipitation experiments from HEK293 cells co-expressing NT^WT^/NT^A77T^/NT^R169L^/NT^G357S^ with HA-tagged RyR2-CT (human RyR2 residues 3529-4967). HA-RyR2-CT was immunoprecipitated with Ab^HA^, verified by Western blotting (Figure 4A, bottom panel), while the presence of co-precipitated NT was analyzed by Western blotting using Ab^cMyc^ (Figure 4A, top panel). NT^WT^ and mutants were recovered in the HA immunoprecipitate but not in the negative control with non-immune rabbit IgG. Cumulative data (Figure 4B) indicate that immunoprecipitation of HA-RyR2-CT resulted in co-precipitation of NT^R169L^ to levels equivalent to NT^WT^, which is consistent with our previous finding that deletion of the β8-β9 loop is dispensable for the interaction between the RyR2 N- and C-termini (Seidel et al., 2021). In contrast, the type II^inter^ A77T mutation significantly enhanced NT interaction with RyR2-CT. Rather surprisingly, the type II^intra^ variant, G357S, did not alter NT interaction with RyR2-CT. To test whether this is the case with other pro-arrhythmic mutations located within the NTD-CSol intra-subunit contact, we studied the R407I variant (Tester et al., 2012) (Table 1). Similar to G357S, we found no difference in NT^R407I^ interaction with RyR2-CT nor in NT^R407I^ tetramerization compared to WT (data not shown).

### 3.2 A77T and R169L alter distinct cellular Ca^2+^ handling properties

**FIGURE 5:**
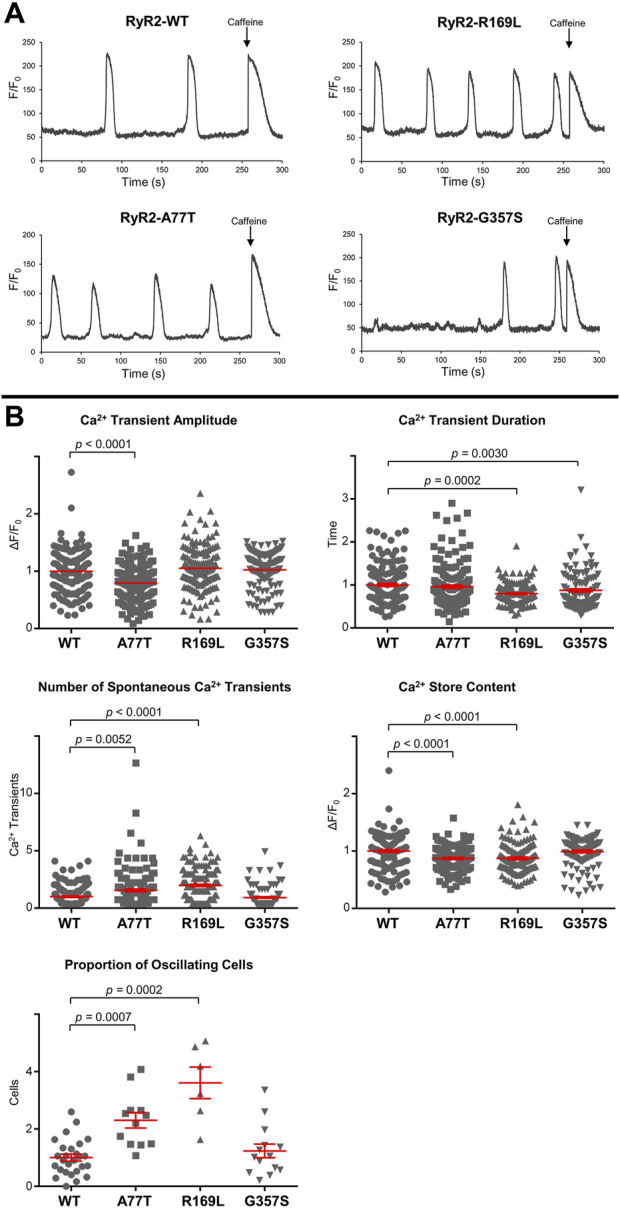
RyR2^A77T^ and RyR2^R169L^ display gain-of-function channel characteristics. Single cell Ca^2+^ imaging using confocal laser scanning microscopy to monitor spontaneous Ca^2+^ release transient events. **(A)** Fluorimetric traces from Cal-520 loaded single HEK293 cells expressing RyR2^WT^, RyR2^A77T^, RyR2^R169L^ or RyR2^G357S^ showing spontaneous Ca^2+^ transients and Ca^2+^ release induced by caffeine (10 mM) at the end of each experiment. **(B)** Cellular Ca^2+^ handling properties including spontaneous Ca^2+^ transient amplitude and duration, number of spontaneous Ca^2+^ transient events, amplitude of the caffeine response (Ca^2+^ store content) and proportion of oscillating cells. Data (for n ≥ 119 cells from n ≥ 6 separate experiments) are normalized for WT and given as mean value ± SEM (shown in red for clarity); statistical analysis was carried out using Kruskal–Wallis test with Dunn’s multiple comparison test.

To assess RyR2 Ca^2+^ release properties, we fluorimetrically monitored spontaneous Ca^2+^ oscillations in HEK293 cells expressing RyR2^WT^/RyR2^A77T^/RyR2^R169L^/RyR2^G357S^ using single cell Ca^2+^ imaging. At the end of the recording, cells were challenged with 10 mM caffeine to verify expression of functional RyR2 channels and estimate the Ca^2+^ store content. Ca^2+^ handling parameters of RyR2^G357S^-expressing cells were similar to RyR2^WT^. Although Ca^2+^ transient duration was altered, the proportion of oscillating cells, number of spontaneous Ca^2+^ transients, and the Ca^2+^ store content were not significantly different to WT (Figure 5). On the other hand, R169L significantly increased the number of spontaneous Ca^2+^ transients as well as the proportion of oscillating cells, indicating that this mutation is hyperactive. The amplitude of spontaneous Ca^2+^ transients was unaffected, whereas their duration was smaller in RyR2^R169L^-expressing cells. The caffeine-induced Ca^2+^ transient amplitude used as an index of Ca^2+^ store content was reduced, suggesting that RyR2^R169L^ is a leaky channel. These characteristics are almost identical to another gain-of-function type I variant, R176Q, which we have previously characterized (Seidel et al., 2021). Similar to R169L, A77T also significantly increased the number of spontaneous Ca^2+^ transients as well as the proportion of oscillating cells, and decreased Ca^2+^ store content indicating that this mutation is also hyperactive and leaky. However, in contrast to the type I variants R169L and R176Q (Seidel et al., 2021), Ca^2+^ transient amplitude was smaller, whereas Ca^2+^ transient duration was unaffected in RyR2^A77T^-expressing cells (Figure 5). Thus, the type II^inter^ A77T mutation affects Ca^2+^ handling parameters differently to type I variants, consistent with the presence of a different underlying molecular mechanism for channel deregulation.

## 4 Discussion

It has long been recognized “that a single mechanism is unlikely to operate in all (RyR2) mutations” (Venetucci et al., 2012). We have very recently suggested that three non-mutually exclusive molecular mechanisms are at play for gain-of-function RyR2 mutations, namely, 1. altered cytosolic and/or luminal Ca^2+^ activation, 2. altered RyR2 intra- and inter-subunit interactions, and 3. altered interactions with accessory proteins (Fowler and Zissimopoulos, 2022). Quite often though, different mechanisms are proposed by different laboratories, whereas contradicting results have been reported even for the exact same mutation. In the present study, we investigated alternative molecular mechanisms for RyR2 mutations within the same structural (NTD) domain. Notably, our investigations were informed by the high-resolution RyR1/2 3D structure in the open and closed configuration (des Georges et al., 2016; Peng et al., 2016) (Table 1).

The type I NTD-NTD inter-subunit interface is largely comprised of the β8-β9 loop (residues 165-179) in one subunit closely apposed to the β23-β24 loop (residues 395-402) of a neighboring subunit (Tung et al., 2010; Peng et al., 2016; Seidel et al., 2021; Zhang et al., 2022). We have previously shown that the arrhythmogenic mutation L433P perturbs both RyR2 N-terminus and full-length tetramerization to produce functionally aberrant channels with both hyperactive and hypoactive characteristics (Seidel et al., 2015). Given that leucine-433 is buried within the NTD structure (Kimlicka et al., 2013; Peng et al., 2016), proline substitution is likely to induce local conformational changes indirectly affecting the NTD-NTD inter-subunit interface. More recently, we and others provided evidence that the CPVT mutations A165D, R169Q and R176Q that lie within the β8-β9 loop, disrupt RyR2 N-terminus self-association to produce hyperactive and leaky channels (Xiong et al., 2018; Nozaki et al., 2020; Seidel et al., 2021; Zhang et al., 2022). In this study, we find that the type I R169L variant perturbs NTD tetramerization, whereas it has no impact on the NTD-CSol interaction (Figures 3, 4), in agreement with our previous findings for the R169Q, R176Q and β8-β9 loop deletion mutants (Seidel et al., 2021; Zhang et al., 2022). Strikingly, Ca^2+^ handling parameters of RyR2^R169L^-expressing cells (Figure 5) are very much alike our previous observations with the hyperactive R176Q mutation (Seidel et al., 2021).

It is clear from the RyR1/2 3D structures that the CSol serves as the primary transducer of cytoplasmic long-range allosteric signals to the channel domain (des Georges et al., 2016; Peng et al., 2016). It is likely that NTD-NTD and NTD-CSol inter-domain interactions act synergistically to regulate channel gating, as revealed by the high-resolution 3D structure of RyR1-R164C. R164C, a malignant hyperthermia mutation within the β8-β9 loop, produced a localized shift and rotation within the NTD resulting in weaker NTD-NTD inter-subunit interaction (Iyer et al., 2020). This in turn further stabilized the NTD-CSol contact, thus conferring to the channel a conformation between fully opened and closed. We have recently reported that the R420Q mutation enhances NTD-CSol interaction (Yin et al., 2021), however, its effect is indirect because this residue is buried within the NTD structure (Kimlicka et al., 2013; Peng et al., 2016). To certify that altered NTD-CSol interaction is indeed a mechanistic basis of RyR2 deregulation, we sought to characterize arrhythmogenic variants that lie at the NTD-CSol intra- (G357S, R407I) and inter-subunit interfaces (A77T) (Table 1).

Our biochemical studies indicate that the type II^intra^ G357S and R407I mutations do not affect NTD tetramerization (Figure 3), in agreement with their location on the RyR2 homotetrameric structure (Peng et al., 2016). However, they also do not seem to affect the NTD-CSol interaction (Figure 4), which is rather unexpected given their location at the intra-subunit interface (Peng et al., 2016) (Table 1). It is plausible that type II^intra^ mutations may result in structural alterations that are beyond the detection limit of our biochemical assays. An alternative explanation is that G357S and R407I (and possibly other type II^intra^ mutations) cause minimal perturbation to the global RyR2 structure. Notably, the Ca^2+^ handling properties of RyR2^G357S^-expressing cells were equivalent to WT (Figure 5), suggesting that this mutation on its own is not sufficient to perturb RyR2 function. Liu *et al.* reported that the G357S mutation reduced the percentage of RyR2-expressing cells that displayed spontaneous Ca^2+^ oscillations, but it increased fractional Ca^2+^ release in individual cells (Liu et al., 2017). These findings may be partly due to reduced RyR2^G357S^ protein expression in their experimental setup. On the other hand, Liu and co-workers found no change in the sensitivity to Ca^2+^ activation and no alteration in the Ca^2+^ store content (Liu et al., 2017). Similarly, an earlier study by Wangüemert and colleagues reported no differences in the caffeine sensitivity or the propensity for spontaneous Ca^2+^ oscillations between RyR2^G357S^ and RyR2^WT^ (Wanguemert et al., 2015), in accordance with our findings. Interestingly, Wangüemert *et al.* found enhanced caffeine sensitivity and an increase in spontaneous Ca^2+^ oscillations for RyR2^G357S^ compared to WT, following treatment with forskolin to increase intracellular cAMP levels (Wanguemert et al., 2015). Thus, it seems that an additional trigger in the form of catecholaminergic stress is required for the G357S mutation to produce defective RyR2 channels.

Unlike type II^intra^ mutations, we find that the type II^inter^ A77T variant enhances NTD-CSol interaction without affecting NTD tetramerization (Figures 3, 4), which is entirely consistent with its location on the RyR2 homotetrameric structure (Peng et al., 2016). Functionally, A77T produces a hyperactive and leaky RyR2 channel, but affecting different Ca^2+^ release parameters to type I mutations, R169L and R176Q (Figure 5 and (Seidel et al., 2021), respectively). These findings are consistent with a largely more compact NTD-CSol inter-subunit interface, dominated by the R76-D3887, E80-W3890 and R111-N4008 interacting pairs, in the open compared to the closed RyR2 configuration (Peng et al., 2016) (Table 1). The RyR2 A77T mutation has been reported as a potential cause in a case of adult-onset genetic generalized epilepsy, where the patient never described any cardiac symptoms and had repeatedly normal cardiac investigations including normal exercise stress tests (Yap and Smyth, 2019). However, the patient’s sibling was diagnosed with CPVT, whereas alanine-77 substitution by Val has also been linked with CPVT (d’Amati et al., 2005). Thus, increased RyR2 NTD-CSol inter-subunit interaction may be a mechanism that underpins neurocardiac calcium channelopathies manifesting as CPVT and/or epilepsy.

**FIGURE 6:**
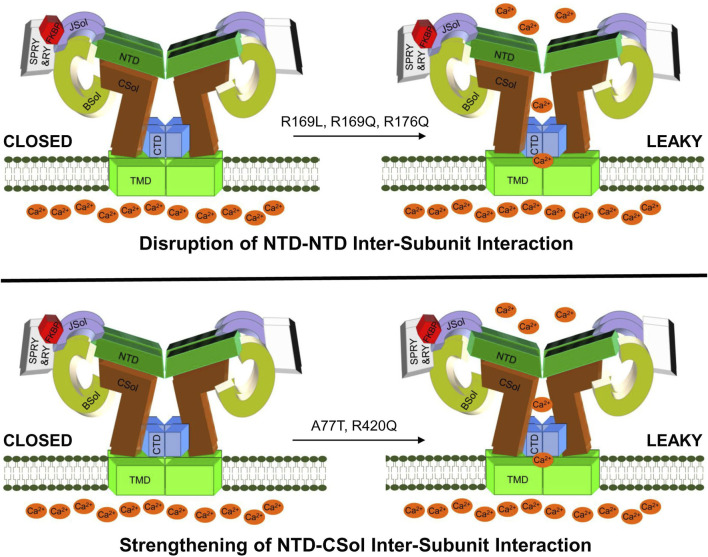
A hypothetical model for deregulated RyR2 in CPVT. Drawing depicting proposed altered RyR2 inter-domain interactions due to NTD gain-of-function mutations. CPVT mutations may produce hyperactive leaky RyR2 channels by disrupting the NTD-NTD inter-subunit interaction (e.g., R169L, R169Q, R176Q) or by strengthening the NTD-CSol inter-subunit interaction (e.g., A77T, R420Q).

In summary, our findings introduce a novel RyR2 structure-function parameter, namely, the NTD-CSol inter-subunit interaction, in the pathogenesis of arrhythmogenic cardiac disease. Moreover, they consolidate the role of the NTD self-association in RyR2 pathophysiology. Importantly, we provide compelling evidence that pro-arrhythmic RyR2 mutations have diverse molecular mechanisms of action, even though they may be very closely located within the primary peptide sequence and/or the same structural domain (Figure 6). This may explain the variable efficacy of potential anti-arrhythmics like K201 and dantrolene, reported to be dependent on the precise location of the RyR2 mutation (Connell et al., 2020). Further studies are required to enable a comprehensive classification of RyR2 mutations into distinct groups with discrete structural and functional phenotypes, which may aid future personalized medicine efforts.

## Data Availability

The raw data supporting the conclusions of this article will be made available by the authors, without undue reservation.
