# Supplementary figures and images for: Disparate molecular mechanisms in cardiac ryanodine receptor channelopathies
Source: Front Mol Biosci. 2024 Dec 24;11:1505698. doi: 10.3389/fmolb.2024.1505698 (PMC11703740; doi:10.3389/fmolb.2024.1505698)

Original image files

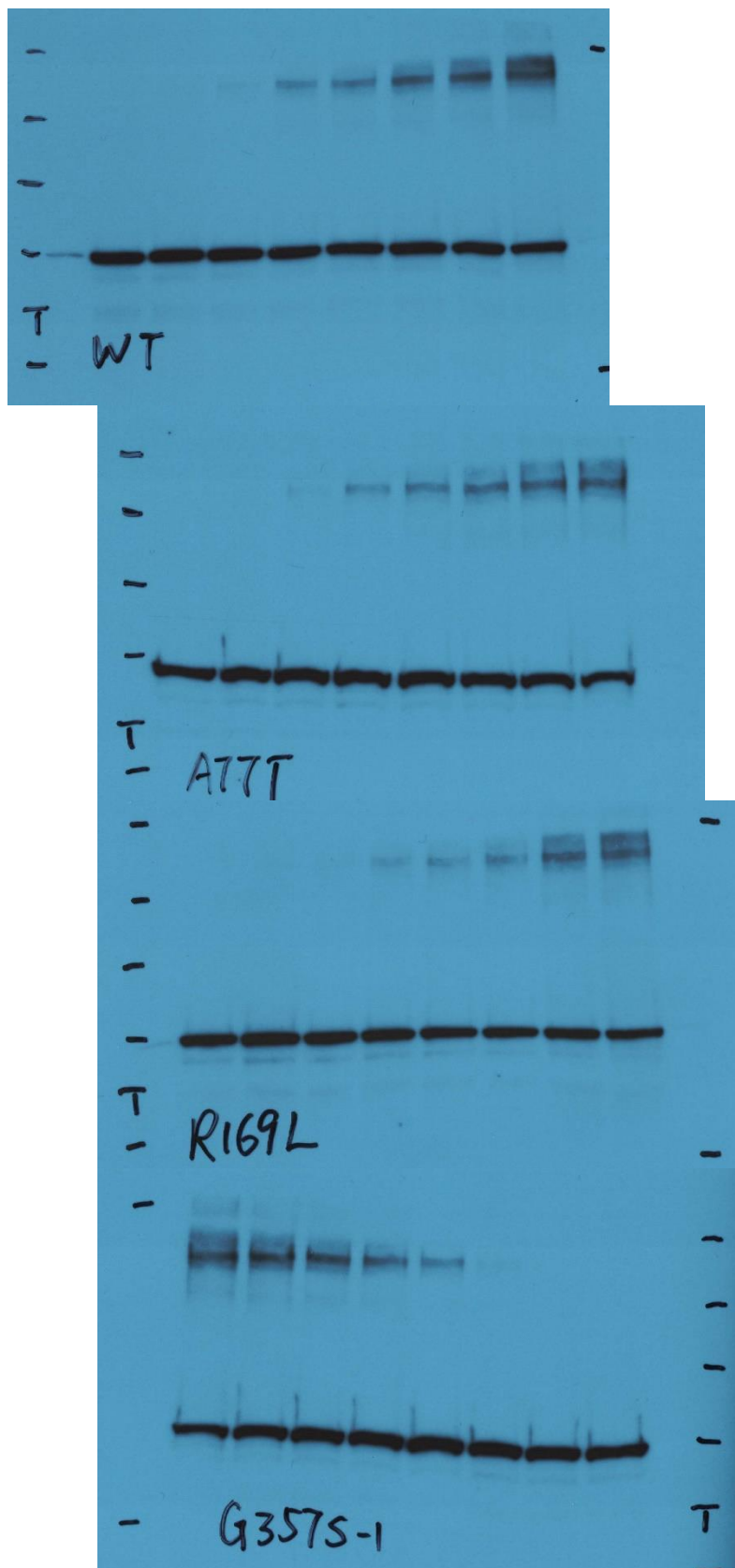

Figure 1

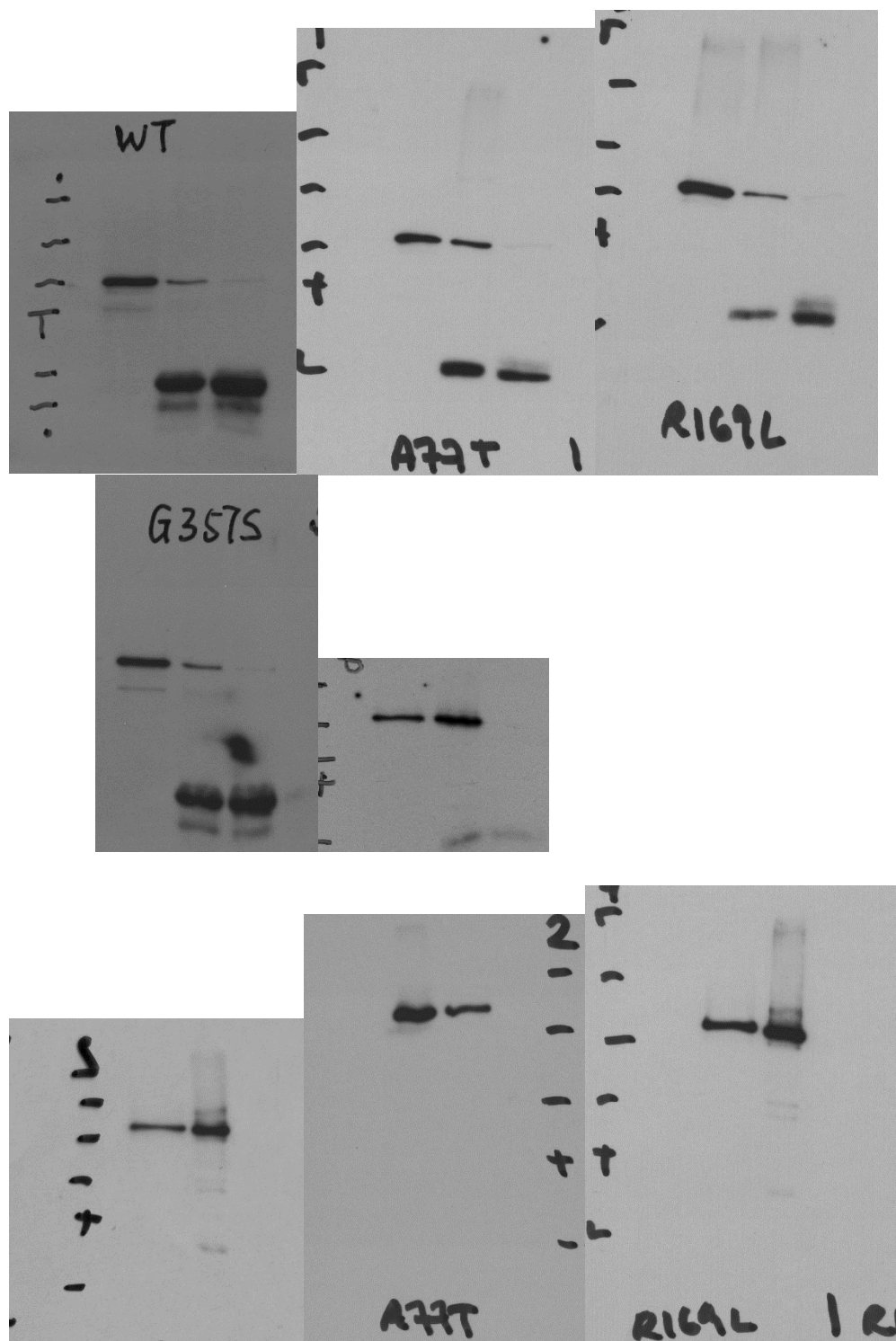

Figure 2

Supplement: Supplementary file 1 [file DataSheet1.pdf]
